# Overexpression of *TaSNAC4-3D* in Common Wheat (*Triticum aestivum* L.) Negatively Regulates Drought Tolerance

**DOI:** 10.3389/fpls.2022.945272

**Published:** 2022-07-04

**Authors:** Jianhui Ma, Mengqi Zhang, Wenming Lv, Xiaoxiao Tang, Dongyang Zhao, Li Wang, Chunxi Li, Lina Jiang

**Affiliations:** College of Life Science, Henan Normal University, Xinxiang, China

**Keywords:** *Triticum aestivum* L., *TaSNAC4-3D*, drought stress, hypersensitive, transgenic wheat

## Abstract

The development and production of bread wheat (*Triticum aestivum* L.) are widely affected by drought stress worldwide. Many NAC transcription factors (TFs) of stress-associated group (SNAC) are functionally proven to regulate drought tolerance. In this study, we identified 41 TaSNACs that were classified into 14 groups, and the expression of *TaSNAC4-3D* was induced in the leaf tissue *via* osmotic or abscisic acid (ABA) treatment. TaSNAC4-3D was localized to the nucleus through the transient expression assay, and the C-terminal region exhibited transcriptional activity *via* transactivation assays. *TaSNAC4-3D* was overexpressed in common wheat. The wheat plants with *TaSNAC4-3D* overexpression was more sensitive to drought stress compared with wild-type (WT) plants. The water loss rate showed no difference between transgenic lines and WT plants. However, drought stress increased H_2_O_2_ and O^2–^ accumulation and promoted programmed cell death (PCD) in the leaf tissue of *TaSNAC4-3D* overexpression lines compared with WT plants. RNA-seq analysis was performed under well-watered and drought conditions, and four strong potential target genes, encoding senescence regulators, were identified by analyzing their promoters containing the NAC recognition sequence (NACRS). Based on these results, our findings revealed that TaSNAC4-3D negatively regulates drought tolerance by inducing oxidative damage in bread wheat.

## Introduction

Drought stress is a common abiotic stress in crop development and yield formation, and drought conditions have become more severe in recent years. Owing to their sessile lifestyle, crops have developed various strategies to cope with the adverse effects by drought stress. Transcriptional regulation is a crucial mechanism that regulates drought response, and many transcription factors (TFs) have been functionally verified to regulate drought tolerance in crops (Fujita et al., 2004; Ma et al., 2017; Li Y. et al., 2019; Wu et al., 2019).

The NAC (i.e., NAM, ATAF, and CUC2) TFs are among the largest TF families, with 105 NAC TFs in *Arabidopsis*, 151 in rice, and 283 in cotton (Ooka et al., 2003; Sun et al., 2018), several of which were functionally verified to regulate drought tolerance. Three AtNAC TFs, namely, ANAC019, ANAC055, and ANAC072, specifically bind to the CATGTG motif and were found to positively regulate drought stress (Tran et al., 2004). The expression of *ATAF1*, an NAC gene in *Arabidopsis*, was induced by drought and ABA treatments, and the *Arabidopsis* plants with *ataf1* mutant showed high tolerance to drought stress (Lu et al., 2007). The AtNTL4, an NAC TF, was found to regulate the expression of several genes for reactive oxygen species (ROS) biosynthesis by binding to their promoters to promote ROS accumulation in *Arabidopsis*, which results in hypersensitivity to drought stress (Lee et al., 2012). ANAC016 inhibits the expression of *AREB1* by binding to its promoter, thereby negatively regulating drought stress in *Arabidopsis* (Sakuraba et al., 2015). OsNAC2 was found to bind to the promoters of *OsLEA3* and *OsSAPK1*, and the knockdown of *OsNAC2* improved the rice yield under drought condition (Shen et al., 2017). *OsNAC066* expression was induced by different abiotic stresses, and the rice plants with *OsNAC066* knockout showed hypersensitivity to drought stress (Wang et al., 2020). OsNAC14 binds to the promoter of *OsRAD51A1* and was proposed to positively regulate drought tolerance *via* DNA damage repair in rice (Sung et al., 2018). In cotton, GhirNAC2 binds to the promoters of *GhNCED3*s, which are responsible for ABA biosynthesis, and cotton plants with *GhirNAC2*s co-suppression are drought-sensitive (Shang et al., 2020). The expression of *GhJUB1L1*, a NAC gene in cotton, was induced by drought stress, and silencing it reduced drought tolerance (Chen Q. et al., 2021). Furthermore, many drought-responsive NAC TFs were functionally identified in tomatoes, maize, soybeans, and other plants (Peng et al., 2009; Mao et al., 2015; Tak et al., 2017; Nguyen et al., 2018; Thirumalaikumar et al., 2018). The aforementioned studies proved the critical roles of NAC TFs in regulating drought tolerance.

As a main food crop, bread wheat (*Triticum aestivum* L.) is the primary source of food for 30–40% of the population (Ray et al., 2013). In recent years, drought frequency and severity have worsened greatly affecting wheat production. Daryanto et al. (2016) examined wheat yield in response to drought stress using the field experiment data and found that drought stress induced a reduction of approximately 21% in wheat yield from 1980 to 2015. Therefore, screening of drought-responsive genes is crucial for further molecular breeding. Many TaNACs have been found to regulate drought response (Ma et al., 2022). *TaSNAC4-3A* expression is induced by drought stress, and its overexpression in *Arabidopsis* significantly increased drought tolerance by regulating stomatal aperture (Mei et al., 2021). *TaNAC69* was overexpressed in wheat, and the transgenic wheat plants showed a higher biomass compared with WT plants under drought stress (Xue et al., 2011). *TaSNAC8-6A* overexpression in wheat stimulated the development of lateral roots to enhance drought tolerance, and chip-seq revealed several targeted genes of TaSNAC8-6A involved in auxin signaling pathways (Mao et al., 2020). TaNAC47 could bind to the ABA-responsive element (ABRE) *cis*-element, and the *Arabidopsis* plants with *TaNAC47* overexpression showed hypersensitivity to ABA and higher tolerance to drought stress (Zhang L. et al., 2016). *TaNAC071-A* was overexpressed in wheat and was found to positively regulate drought tolerance modulated by TaMYBL1 (Mao et al., 2021). In our previous study, we collected a total of 12 drought-responsive TaNACs; interestingly, 10 of them were TaSNACs (Ma et al., 2022). Therefore, analyzing and selecting drought-responsive TaSNACs is of great significance. In this study, we identified 41 TaSNACs *via* genome-wide analysis by combining the previous studies and characterized a new drought-responsive NAC transcription factor of stress-associated group from wheat (TaSNAC) gene, named *TaSNAC4-3D*, which was found to negatively regulate drought tolerance by increasing oxidative damage in transgenic wheat plants with *TaSNAC4-3D* overexpression.

## Materials and Methods

### Plant Materials and Treatment

The wheat *cv.* Chinese Spring was used for expression analysis, and wheat seedlings were cultured as described by Ma et al. (2022). At the two-leaf stage, the wheat seedlings were transferred into Hogland solution and were then divided into control and Hogland solution containing 15% PEG-6,000 and 100 μM abscisic acid (ABA) treatment groups, respectively. The leaf and root tissues from the control and treated wheat seedlings were collected with three biological replications for each time point. For drought stress in soil, wheat seedlings were cultured as described by Ma et al. (2022). The leaf tissue was sampled at the point of relative water content (RWC) of soil of 50 and 40% with three biological replications, and the well-watered samples were regarded as the control group. All samples were stored at –80°C until RNA extraction.

### Cloning of *TaSNAC4-3D*, Phylogenetic Tree Construction, and Gene Structure Analysis

The specific primers for *TaSNAC4-3D* cloning were designed using the genome data (Supplementary Table 1), and the PCR product was inserted into the pMD18-T vector (TaKaRa, Japan). The plasmids of the positive clones were sequenced to confirm the sequence of *TaSNAC4-3D* (Sangon, Shanghai, China). The full-length protein sequences of selected TaSNACs were used for multiple alignment using ClustalW, and a phylogenetic tree was constructed using the MEGA 5.11 software by the neighbor-joining method (Tamura et al., 2021), and bootstrap analysis was performed for 1,000 replicates. The gene structure of TaSNACs was determined using the Gene Structure Display Server 2.0 (GSDS, Hu et al., 2015).

### RNA Extraction and qRT-PCR Analysis

TRIzol (TaKaRa, Japan) reagent was used for total RNA extraction, which was further used for first-strand cDNA synthesis using a PrimeScript RT reagent kit with gDNA Eraser (TaKaRa, Japan). The specific primers were designed for qPCR (Supplementary Table 1) on an ABI 7500 real-time PCR system (Applied Biosystems, United States) using the SYBR Premix Ex Taq Kit (TaKaRa, Japan). As the internal control, the wheat *Tubulin* gene was used to calculate the expression level by the 2^–ΔΔCt^ method.

### Transcriptional Activity and Subcellular Localization Analysis of TaSNAC4-3D

The TaSNAC-N (1–160 aa) and TaSNAC-C (161–316 aa) sequences were confirmed according to the location of the NAM domain, and primers were designed (Supplementary Table 1). Transcriptional activity assay was performed as described previously (Ma et al., 2022). For the subcellular localization assay, the specific primers without stop codons were used to obtain the complete coding sequence (CDS) of *TaSNAC4-3D*, and the product was inserted into the pJIT163-GFP vector to construct recombinant plasmids between the *Hind*III and *BamH*I sites. The recombinant plasmid and control pJIT163-GFP vectors were transformed into *Arabidopsis* protoplasts, which were prepared using the *Arabidopsis* Protoplast Preparation and Transformation Kit (Coolaber, Beijing, China). Green fluorescent protein (GFP) fluorescence was monitored using a laser scanning confocal microscopy (TCS SP8, Leica, Germany).

### Generation of Transgenic Wheat Plants With *TaSNAC4-3D* Overexpression

To obtain *TaSNAC4-3D* transgenic wheat plants, the complete CDS of *TaSNAC4-3D* was inserted into the pMWB110 vector. An *Agrobacterium*-mediated transformation system was used to introduce genetic transformation of *TaSNAC4-3D* in bread wheat (*cv.* Fielder). PCR amplification and qRT-PCR were performed to screen transgenic plants (Supplementary Table 1). Homozygous T3 transgenic seeds were obtained for further analysis.

### Determination of Fresh Weight, Dry Weight, Malondialdehyde Content, Proline Content, Water Loss Rate, H_2_O_2_ Content, O^2–^ Content, and Soil RWC

The aboveground part of each plant under drought condition was weighed to evaluate the fresh weight and dried in an oven at 80°C for 24 h for dry weight. Each experiment included six replications. About 0.3 g of leaf tissue for each sample was used to determine the malondialdehyde (MDA) content (Zheng et al., 2008), which was determined by the absorption values at 600, 532, and 450 nm. For the determination of the proline content, about 0.3 g of leaf tissue for each sample was homogenized with 3% (w/v) sulfosalicylic acid, which was centrifuged at 10,000 g for 5 min. The supernatant (2 ml) was mixed with 2 ml of glacial acetic acid and 2 ml of acid ninhydrin, which was incubated in boiling water for 60 min. After cooling in ice, 4 ml of toluene was added to the mixture to analyze the absorbance of the chromophore. A calibration curve was established to determine the profile content using an UV spectrometer (UV-2600, Shimadzu, Kyoto, Japan) at 520 nm (Bates et al., 1973). Notably, 10 leaves from each line under well-watered condition were used for the determination of the water loss rate as described by Mega et al. (2019) and as described in our previous study (Ma et al., 2022). The O^2–^ content and H_2_O_2_ content were determined using the detection kits (Solarbio, Beijing, China), which have been used in a previous study (Chen J. et al., 2021). Soil RWC was measured as our previous description (Ma et al., 2022). All data were obtained from three biological replications.

### Terminal Deoxynucleotidyl Transferase-Mediated dUTP Nick-End Labeling Assay

The wild-type (WT) and transgenic leaves under drought condition were dehydrated in paraffin, and the terminal deoxynucleotidyl transferase-mediated dUTP nick-end labeling (TUNEL) was performed using the TUNEL assay kit, as described by Li Y. B. et al. (2019). The fluorescence microscope at 590 nm was used to obtain photograph.

### RNA-Seq Analysis

The leaves with *TaSNAC4-3D* overexpression and that of WT plants were used for RNA-seq analysis, and total RNA was extracted. Each library was constructed using the NEBNext^®^ Ultra™ RNA Library Prep Kit for Illumina^®^ (NEB, Ipswich, United States) and sequenced on an Illumina HiSeq-PE150. After removing low-quality reads, the clean reads were used for alignment with the genome data of IWGSC RefSeq v1.1 (International Wheat Genome Sequencing Consortium (IWGSC), 2018) by Hisat 2 (version 2.0.5). Differential expression analysis was performed using DEseq2. The raw data were submitted to NCBI with the BioProject ID of PRJNA838079.

## Results

### Genome-Wide Identification of TaSNAC From *Triticum aestivum*

The high-confidence protein database of wheat genome (IWGSC RefSeq v2.1) was used for a Hidden Markov Model (HMM) searching for the NAC TFs (TaNACs, PF02365) based on the HMM profile (Mistry et al., 2021). Totally, 452 TaNACs were identified by further confirmation in SMART platform (Letunic et al., 2021). Detailed information on the 452 TaNACs, including annotation and ID conversion, is provided in Supplementary Table 2 (International Wheat Genome Sequencing Consortium (IWGSC), 2014; Clavijo et al., 2017; International Wheat Genome Sequencing Consortium (IWGSC), 2018). Similar to the previous study, NAC TFs were divided into several groups, including SNAC, ANAC34, NEO, SND, NAC22, NAC1, NAM/CUC3, OMNAC, and TIP (Nuruzzaman et al., 2010). To obtain the TaSNACs, the protein sequences of 452 TaNACs and 12 NACs from *Arabidopsis* for subgroup classification were used to construct a phylogenetic tree. From this phylogenetic tree, 40 TaSNACs were identified (Supplementary Figure 1 and Supplementary Table 3). After a sequence analysis with TaSNACs reported by Mao et al. (2020), TaSNAC8-6B was deleted as no NAM domain was found after HMM searching, and TaSNAC4-3A, which has been functionally verified (Mei et al., 2021), was collected to our research resulting in a total of 41 TaSNACs.

**FIGURE 1:**
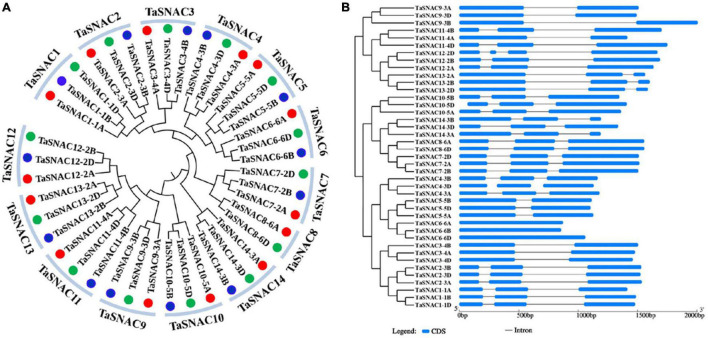
Phylogenetic tree and gene structure analysis of TaSNACs. **(A)** A total of forty-one TaSNACs were classified into 14 homologous groups, TaSNAC1 to TaSNAC14, through phylogenetic tree analysis. **(B)** The coding and genome sequences of 41 TaSNACs were obtained from the genome database and were used for gene structure analysis in the GSDS platform.

A phylogenetic tree was constructed using the 41 TaSNACs, which were divided into 14 homologous groups (Figure 1A). To avoid confusion with the further studies on TaSNACs, they were denoted as described by Mao et al. (2020), and three new members joined, namely, TaSNAC14-3A, TaSNAC14-3B, and TaSNAC14-3D (Supplementary Table 3). The exon–intron structure of these 41 TaSNACs was examined using the online GSDS server to understand the evolution of these genes (Hu et al., 2015), and a similar exon–intron structure was observed within the same homologous group of TaSNACs (Figure 1B). The structure of the relevant genes differed between the groups, indicating that these genes may have different functions during their evolution.

### Expression Pattern Analysis of *TaSNACs*

**FIGURE 2:**
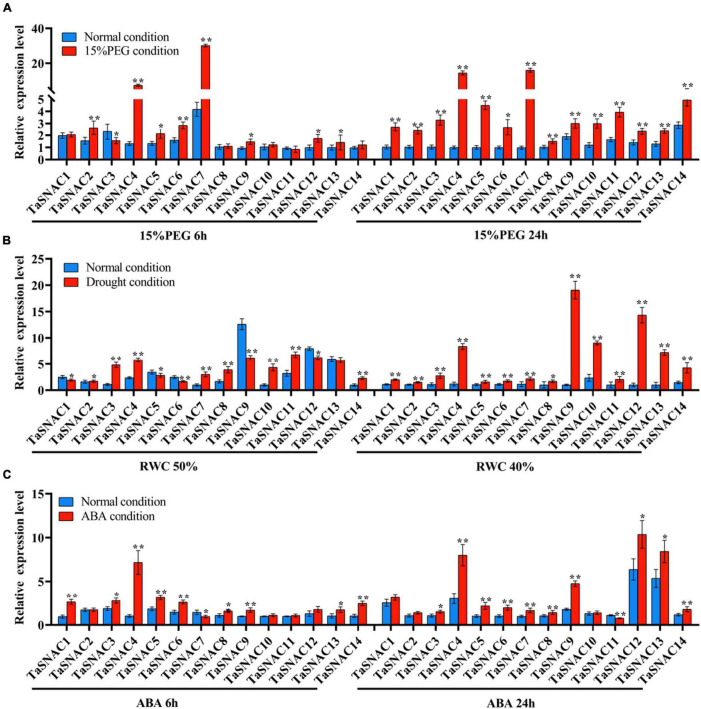
Expression pattern analysis of TaSNACs in 14 homologous groups under osmotic, drought, and abscisic acid (ABA) treatments. Wheat seedlings at the two-leaf stage were subjected to osmotic stress with 15% PEG-6000 for 6 and 24 h, and the expression pattern of 14 groups of TaSNACs was examined in the leaf tissue **(A)**. Wheat seedlings at the two-leaf stage were subjected to drought stress by discontinuing watering, and the expression patterns of 14 groups of TaSNACs were examined in the leaf tissue when the soil RWC was 50 and 40% **(B)**. Wheat seedlings at the two-leaf stage were subjected to 100 μM ABA treatment for 6 and 24 h, and the expression patterns of 14 groups of TaSNACs were examined in the leaf tissue **(C)**. The value is shown as mean ± SD. The *t*-test was performed to analyze the difference between treatment and corresponding control, and the asterisk indicates a significant difference (*P < 0.05; **P < 0.01).

We examined the expression patterns of 14 groups of TaSNACs under 15% PEG-6000, drought, and ABA treatments. Eight groups of TaSNACs were upregulated under 15% PEG-6000 for 6 and 24 h (Figure 2A), seven groups of TaSNACs were upregulated under drought treatment with 50 and 40% soil RWC (Figure 2B), and eight groups of TaSNACs were upregulated under 100 μM ABA treatment for 6 and 24 h (Figure 2C). More importantly, we found that the group of *TaSNAC4* was upregulated with more than 2-fold changes under the three treatments, which got our attention.

**FIGURE 3:**
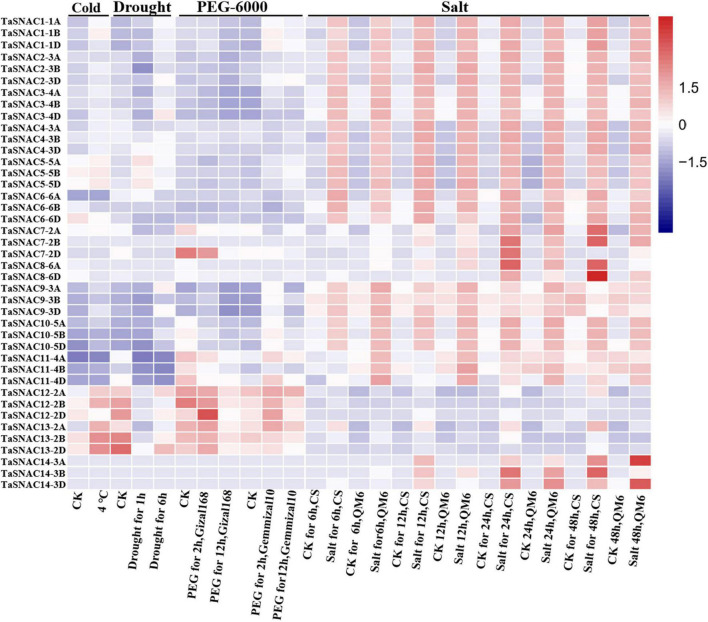
Gene expression heatmap of 41 TaSNACs. The transcriptomic data of abiotic stress, including cold, drought, osmotic, and salt stresses, was used to analyze the expression pattern of 41 TaSNACs, and the fragments per kilobase million value was used to represent the gene expression level. The red color and blue color represent high and low transcript abundance, respectively.

The WheatOmics platform collected the RNA-seq data about wheat (Ma S. et al., 2021), and the RNA-seq data of cold (Li et al., 2015), drought (Liu et al., 2015), osmotic (PRJNA306536), and salt (Zhang Y. et al., 2016) stresses was used to analyze the expression pattern of 41 TaSNACs. Differentially expressed TaSNACs were identified by a 1.5-fold change between the treatment and corresponding control. Among them, 15 differentially expressed TaSNACs were downregulated under cold stress. Under drought stress, 31 differentially expressed TaSNACs were identified, among which 9 TaSNACs were upregulated. After PEG-6000 treatment using *cv.* Giza168 and Gemmiza10, 22 differentially expressed TaSNACs in Giza168 and 30 differentially expressed TaSNACs in Gemmiza10 were identified. Under salt stress using *cv.* Chinese spring and QM6, 28 differentially expressed TaSNACs, including 22 downregulated TaSNACs, were identified in Chinese spring; 28 differentially expressed TaSNACs, including 23 downregulated TaSNACs, were identified in QM6. We found that *TraesCS3D03G0748100* (*TaSNAC4-3D*) was differentially expressed under all abiotic stresses (Figure 3). Therefore, *TaSNAC4-3D* was selected as the candidate gene for further functional analysis.

### *TaSNAC4-3D* Is Upregulated Under Osmotic or Abscisic Acid Treatment

**FIGURE 4:**
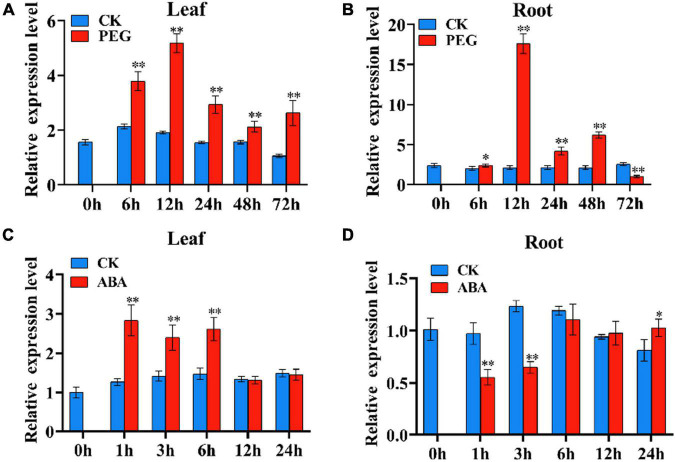
The expression pattern of *TaNAC4-3D* under osmotic stress and ABA treatment in wheat seedlings. The wheat seedlings were subjected to osmotic stress using 15% PEG-6000 for 6, 12, 24, 48, and 72 h at the two-leaf stage, and the corresponding controls were cultured in Hoagland solution. Under osmotic stress, the expression pattern of *TaNAC4-3D* was analyzed in the leaf **(A)** and root **(B)** tissues. The wheat seedlings at the two-leaf stage were cultured in Hoagland solution with 100 μM ABA and Hoagland solution as the control, respectively, for 1, 3, 6, 12, and 24 h. For ABA response, the expression pattern of *TaNAC4-3D* was analyzed in the root **(C)** and leaf **(D)** tissues. The value is shown as mean ± SD. The *t*-test was performed to analyze the difference between treatment and corresponding control, and the asterisk indicates a significant difference (*P < 0.05; ^**^P < 0.01).

The expression pattern of *TaSNAC4-3D* under osmotic and ABA treatments was characterized *via* qRT-PCR. We used 15% PEG-6,000 to simulate osmotic stress. *TaSNAC4-3D* was significantly upregulated in the leaf tissue from 6 to 72 h of osmotic stress (Figure 4A) and in the root tissue from 12 to 48 h of drought stress (Figure 4B). The transcript levels of *TaSNAC4-3D* peaked at 12 h. Under ABA treatment, *TaSNAC4-3D* was upregulated in the leaf tissue for the first 6 h (Figure 4C) and downregulated in the root tissue for the first 3 h (Figure 4D). Therefore, we speculated that *TaSNAC4-3D* might participate in drought response in wheat.

### TaSNAC4-3D Encodes a Nuclear Localized Protein and Exhibits Transcriptional Activity

**FIGURE 5:**
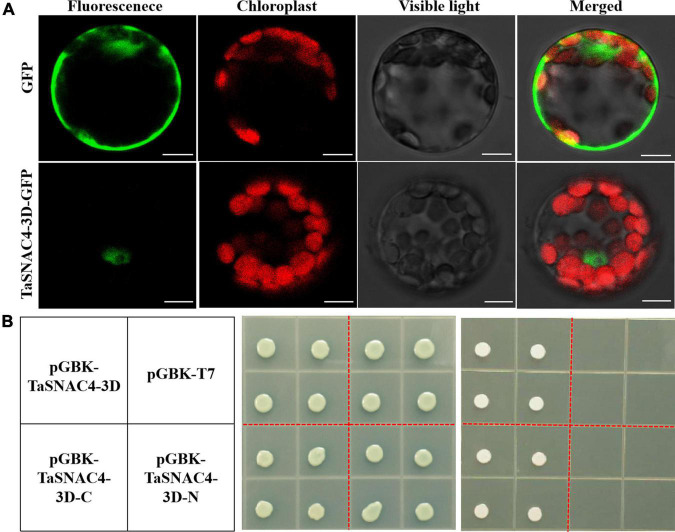
Subcellular localization and transcriptional activity analysis of TaSNAC4-3D. The recombinant plasmid and the control pJIT163-GFP vector were transformed into *Arabidopsis* protoplasts, and TaSNAC4-3D is localized to the nucleus **(A)**. Scale bar = 10 μm. Three recombinant plasmids and empty pGBKT7 vector were transformed into the yeast strain. The growth status of transformed yeasts on SD/-Trp and SD/-Trp/-His/-Ade medium was observed, respectively **(B)**.

TaSNAC4-3D-GFP was transiently expressed in *Arabidopsis* protoplasts to investigate the subcellular localization of TaSNAC4-3D. GFP fluorescence was distributed throughout the cytoplasm and nucleus in the *Arabidopsis* protoplasts harboring control pJIT163-GFP vectors, and the red fluorescence was distributed on the vacuole membrane (Figure 5A). However, GFP fluorescence was specifically observed on the nucleus in *Arabidopsis* protoplasts when the fusion protein of TaSNAC4-3D-3D was expressed (Figure 5A), which confirmed that TaSNAC4-3D is localized to the nucleus.

For transcriptional activity analysis of TaSNAC4-3D, the plasmids of pGBKT7-TaSNAC4-3D, pGBKT7-TaSNAC4-3D-N (1–160 aa), pGBKT7-TaSNAC4-3D-C (161–316 aa), and empty pGBKT7 vector (negative control) were transformed into yeast (AH109), respectively. The transformants grew well on the SD medium without tryptophan (SD/-Trp), indicating the successful transformation of four vectors (Figure 5B), while only the transformants with the pGBKT7-TaSNAC4-3D and pGBKT7TaSNAC4-3D-C grew well on the SD medium without tryptophan, histidine, and adenine (SD/-Trp-His-Ade). These results suggested that the transactivation domain is located at the C-terminus of TaSNAC4-3D.

### Overexpression of *TaSNAC4-3D* Reduced Drought Tolerance in Wheat Seedlings

**FIGURE 6:**
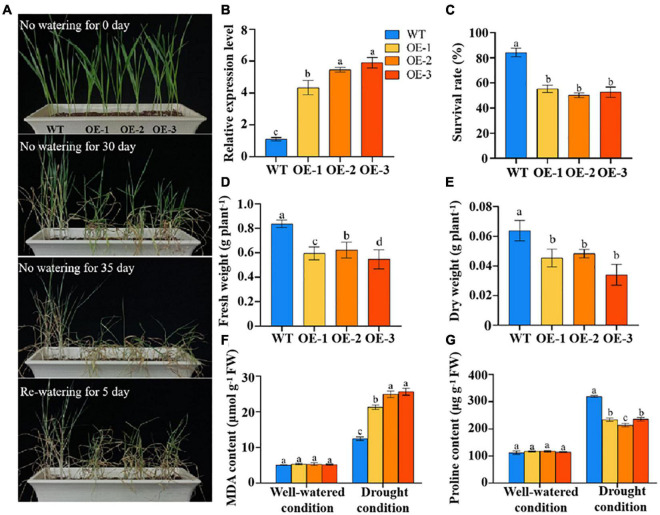
Overexpression of *TaSNAC4-3D* negatively regulates drought tolerance. The phenotype of three transgenic lines and wild-type (WT) plants under drought stress **(A)**. The expression level of *TaSNAC4-3D* in three transgenic lines and WT plants **(B)**. The survival rate **(C)**, fresh weight **(D)**, and dry weight **(E)** were measured using the three transgenic lines and WT plants under drought stress. The contents of MDA **(F)** and proline **(G)** were measured using the three transgenic lines and WT plants under well-watered and drought conditions, respectively. The value is shown as mean ± SD. Analysis of variance was used to analyze the difference, and the different letters mean the difference at 0.05 level.

To investigate the functions of TaSNAC4-3D under drought stress, we obtained transgenic wheat plants with *TaSNAC4-3D* overexpression, which were detected by PCR amplification (Supplementary Figure 2). To assess the function of *TaSNAC4-3D* in drought regulation, we selected three independent lines (i.e., OE1, OE2, and OE3) with high transcript levels and WT plants for further analysis (Figures 6A,B). Under well-watered conditions, the growth status of the *TaSNAC4-3D* overexpression lines and WT plants showed no difference. The transgenic and WT plants were subjected to drought stress by discontinuing watering at the two-leaf stage. After 30 days of drought treatment, the *TaSNAC4-3D* overexpression lines exhibited a moderate stress phenotype, and the WT plants grew well. After 35 days of drought treatment, most leaves of the *TaSNAC4-3D* overexpression lines were wilted, whereas WT plants exhibited a moderate stress phenotype. All plants were rewatered on day 36. After 5 days, normal growth could not be restored in the transgenic plants, whereas the WT plants resumed their growth (Figure 6A).

The survival rate, fresh weight, and dry weight were investigated under drought stress, which were higher in WT plants than three transgenic lines (Figures 6C–E). The MDA and profile content showed no difference under well-watered condition, and significant difference under drought condition between the transgenic and WT plants (Figures 6F,G). From these results, we found that *TaSNAC4-3D* overexpression reduced drought tolerance in wheat seedlings.

### Overexpression of *TaSNAC4-3D* Accelerated Oxidative Damage

**FIGURE 7:**
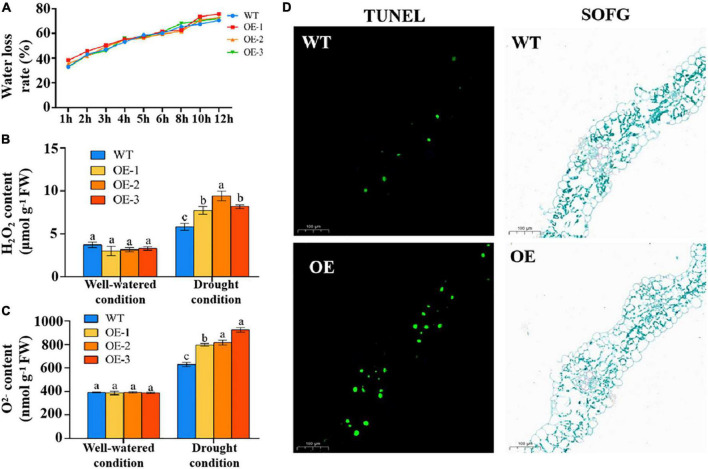
Overexpression of *TaSNAC4-3D* accelerated oxidative damage. The water loss rate was measured using the leaves of three transgenic lines and WT plants **(A)**. The contents of H_2_O_2_ **(B)** and O^2–^ **(C)** were measured using the leaves of three transgenic lines and WT plants under well-watered and drought conditions, respectively. TUNEL staining was performed using the leaves of transgenic and WT plants indicated that overexpression of *TaSNAC4-3D* accelerated PCD under drought stress **(D)**. Scale bar = 100 μm, and FW represents fresh weight. Analysis of variance was used to analyze the difference, and the different letters mean the difference at 0.05 level.

Regulation of water loss is an important physiological process for alleviating drought stress. However, the water loss rate showed no difference between the transgenic lines and WT plants (Figure 7A). Oxidative damage is caused by drought stress. In this study, the contents of H_2_O_2_ and O^2–^ were measured. The two indicators showed no difference between the transgenic lines and WT plants under normal conditions and were significantly higher in the transgenic lines than in WT plants under drought stress (Figures 7B,C). These results suggested that TaSNAC4-3D negatively regulates drought tolerance by increasing oxidative damage in wheat seedlings.

Reactive oxygen species are important signaling molecules for PCD. Therefore, we speculated that PCD might be a factor causing plant wilting. To determine whether wheat leaves undergo PCD under drought stress, TUNEL staining was performed. The results showed that a greater number of green nuclei were observed in transgenic plants compared with WT plants (Figure 7D). These results indicated that *TaSNAC4-3D* overexpression induced excessive production of ROS under drought stress, thereby accelerating PCD.

### RNA-Seq Revealed the Potential Mechanism of TaSNAC4-3D

The above results showed that overexpression of *TaSNAC4-3D* in wheat mediates drought tolerance. To better investigate the potential mechanism underlying the effects of TaSNAC4-3D, we analyzed and compared the RNA-Seq data using the transgenic and WT plants under well-watered and drought conditions. Three independent biological replications were set for each treated sample, and 12 libraries were obtained. The clean reads, average base sequencing error rate, Q20, Q30, and other information regarding these libraries are provided in Supplementary Table 4, and all these data indicate the high quality of 12 libraries.

**FIGURE 8:**
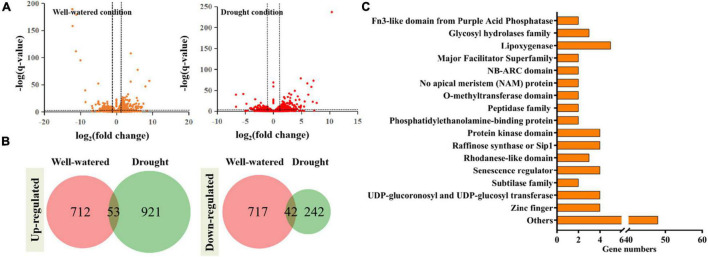
RNA-Seq analysis was performed using the leaves of transgenic and WT plants under well-watered and drought conditions. The DEGs were identified using the parameters: *q*-value < 0.05 and absolute fold change > 2 **(A)**. 53 DEGs were upregulated and 42 DEGs were downregulated under two conditions **(B)**. 93 genes contained the NACRS in their promoters **(C)**.

The differentially expressed genes (DEGs) were selected using the parameters of more than twofold changes and a *q*-value < 0.05. Totally, 1,429 DEGs were obtained from plants with *TaSNAC4-3D* overexpression under well-watered condition (Figures 8A,B), including 712 upregulated and 717 downregulated genes, compared with WT plants, and 1,163 DEGs were obtained from plants with *TaSNAC4-3D* overexpression under drought condition (Figures 8A,B), including 921 upregulated and 242 downregulated genes. Detailed information on gene expression is provided in Supplementary Table 5. Among these DEGs, we found 53 persistently upregulated and 42 persistently downregulated genes under the two conditions (Figure 8B). Functional annotation revealed that the expression of genes, encoding the glycosyl hydrolases family, no apical meristem (NAM) protein, peptidase family, phosphatidylethanolamine-binding protein, protein kinase domain, raffinose synthase or Sip1, rhodanese-like domain, senescence regulator, zinc finger, and others, was upregulated, whereas the expression of genes, encoding fn3-like domain from purple acid phosphatase, lipoxygenase, major facilitator superfamily, NB-ARC domain, O-methyltransferase domain, subtilase family, UDP-glucoronosyl and UDP-glucosyl transferase, and others, was downregulated in the transgenic plants. We further analyzed the promoter of these selected 95 DEGs to find the NACRS with CACG core motif, and 93 persistently DEGs were selected (Supplementary Table 6). Interestingly, the expression of four senescence regulators genes, namely, *TraesCS2A02G222500*, *TraesCS6B02G258900*, *TraesCS6A02G222200*, and *TraesCS6D02G212800*, was upregulated in transgenic plants, and each contained more than ten NACRSs, which may be the strong potential target genes of TaSNAC4-3D.

## Discussion

NAC TFs are plant-specific proteins, and many members were functionally proven to regulate abiotic and biotic stress (Jensen and Skriver, 2014; Kim et al., 2016; Yuan et al., 2019). Many studies performed research on TaNAC identification along with the completion of bread wheat genome sequencing. Totally, 453 TaNACs were identified by Borrill et al. (2017) using the wheat genome by TGAC wheat assembly, and Ma J. et al. (2021) identified 462 TaNACs using IWGS RefSeq v1.0 database. In this study, we identified 452 TaNACs using the latest version of genome database (IWGSC RefSeq v2.1, Supplementary Table 2). This provides important information for further functional analysis. As one of the most important crops, about 50% of bread wheat is grown in arid and semiarid regions, and drought is a major abiotic stress for wheat production (Sternberg, 2011). Therefore, studying the regulatory mechanisms of wheat under drought stress is necessary to further improve its drought resistance. TaNACs were divided into several groups, including SNAC group (Nuruzzaman et al., 2010). In wheat, 12 TaNACs were functionally verified to be involved in drought response; 10 of them were found to be the members of TaSNACs (Ma et al., 2022), including nine positive regulators and one negative regulator. Therefore, TaSNACs are important drought regulators in wheat. A previous study identified 39 TaSNACs using the version of IWGS RefSeq v1.1 database (Mao et al., 2020). In this study, we collected the result of Mao et al. and finally identified 41 TaSNACs, which were divided into 14 groups (Figure 1 and Supplementary Table 3). Through expression pattern analysis, we found that most of them were differentially expressed under abiotic stress, and *TaSNAC4* upregulated more than 2-fold changes under three abiotic stresses (Figure 2). By analyzing the RNA-seq data about abiotic stresses in wheat, *TaSNAC4-3D* showed differentially expressed under all abiotic stresses (Figure 3). Therefore, *TaSNAC4-3D* was selected for functional analysis.

Plants have developed several complex regulatory processes to adapt to drought stress. Under drought stress, water loss is reduced through stomatal closure, and several TaSNACs, including TaNAC2D, TaNAC2, and TaNAC48, were proved to regulate water loss under drought stress (Mao et al., 2012; Huang and Wang, 2016; Chen J. et al., 2021). However, the water loss rate was comparable between *TaSNAC4-3D* overexpression lines and WT plants. We further examined the content of H_2_O_2_ and O^2–^ and found that they were significantly higher in *TaSNAC4-3D* overexpression lines than in WT plants (Figure 7). OsNAC4, a homolog of TaSNAC4-3D in rice, was functionally verified to promote PCD (Kaneda et al., 2009), and ANAC081, a homolog of TaSNAC4-3D in *Arabidopsis*, was proven to promote leaf senescence (Nagahage et al., 2020). These studies indicated that TaSNAC4-3D may be a negatively regulator in response to abiotic stress. However, TaSNAC4-3A, which belongs to the TaSNAC4 group, was found to positively regulate drought tolerance through its overexpression in *Arabidopsis* (Mei et al., 2021). In this study, *TaSNAC4-3D* was overexpressed in wheat, and wheat plants with *TaSNAC4-3D* overexpression were hypersensitive to drought stress (Figure 6). We further found that the transgenic plants accumulated more ROS content than WT plants under drought stress (Figure 7), which functioned similar to that of homologous genes in rice and *Arabidopsis*. Therefore, TaSNAC4-3D was confirmed to negatively regulate drought stress in wheat.

Programmed cell death is an important mechanism to deal with environmental stress (Wu et al., 2016), and excess ROS accumulation usually promotes PCD (Gechev et al., 2012). We observed the significant accumulation of H_2_O_2_ and O^2–^ content in transgenic plants under drought stress compared with WT plants (Figure 7). In addition, overexpression of *TaSNAC4-3D* accelerated PCD under drought stress, which should be closely related to oxidative damage. Researchers have attempted to analyze the potential mechanism of TaNACs using RNA-seq (Mao et al., 2020; Mei et al., 2021), and the NACRS in the promoter was considered to be the binding core of the NAC TFs. In this study, we obtained 95 reliable DEGs using RNA-seq under well-watered and drought growth conditions, of which 93 DEGs contained NACRS in their promoters as the potential target genes (Figure 8 and Supplementary Table 5). As the homolog of TaSNAC4-3D in *Arabidopsis* was found to positively regulate leaf senescence (Nagahage et al., 2020), four upregulated senescence regulators in transgenic plants got our attention. Furthermore, the promoter of each senescence regulator gene contains more than 10 NACRSs (Supplementary Table 6). They should be the strong potential target genes of TaSNAC4-3D and play important roles in negatively regulating drought stress. Further studies are required to elucidate a more detailed mechanism.

In summary, we identified 452 TaNACs and 41 TaSNACs from the latest version of genome database (IWGSC RefSeq v2.1, Supplementary Table 2). From the expression pattern analysis of TaSNAC genes, a drought-responsive TaSNAC, *TaSNAC4-3D*, was selected. *TaSNAC4-3D* was overexpressed in wheat, and the transgenic wheat plants showed hypersensitivity to drought stress compared with WT plants that was possibly induced by oxidative damage. Moreover, four genes encoding senescence regulators were found to be the strong potential target genes of TaSNAC4-3D through RNA-seq and functional analysis.

## Data Availability Statement

The original contributions presented in this study are publicly available. This data can be found here: NCBI, PRJNA838079.

## Author Contributions

JM and LJ conceived and designed the research. JM, MZ, WL, XT, DZ, LW, and CL performed the experiments. JM, MZ, and XT analyzed the data. JM, MZ, XT, and DZ prepared the figures and provided the materials. JM and XT wrote the manuscript. All authors have read and approved the final manuscript.

## Conflict of Interest

The authors declare that the research was conducted in the absence of any commercial or financial relationships that could be construed as a potential conflict of interest.

## Publisher’s Note

All claims expressed in this article are solely those of the authors and do not necessarily represent those of their affiliated organizations, or those of the publisher, the editors and the reviewers. Any product that may be evaluated in this article, or claim that may be made by its manufacturer, is not guaranteed or endorsed by the publisher.

## Supplementary Material

Supplementary Figure 1 The phylogenetic tree using 452 TaNACs and 12 NACs from *Arabidopsis* for TaSNAC identification.

Supplementary Figure 2 Identification of *TaSNAC4-3D* overexpression plants using PCR amplification.
