# Supplementary figures and images for: Overexpression of TaSNAC4-3D in Common Wheat (Triticum aestivum L.) Negatively Regulates Drought Tolerance
Source: Front Plant Sci. 2022 Jul 4;13:945272. doi: 10.3389/fpls.2022.945272 (PMC9289557; doi:10.3389/fpls.2022.945272)

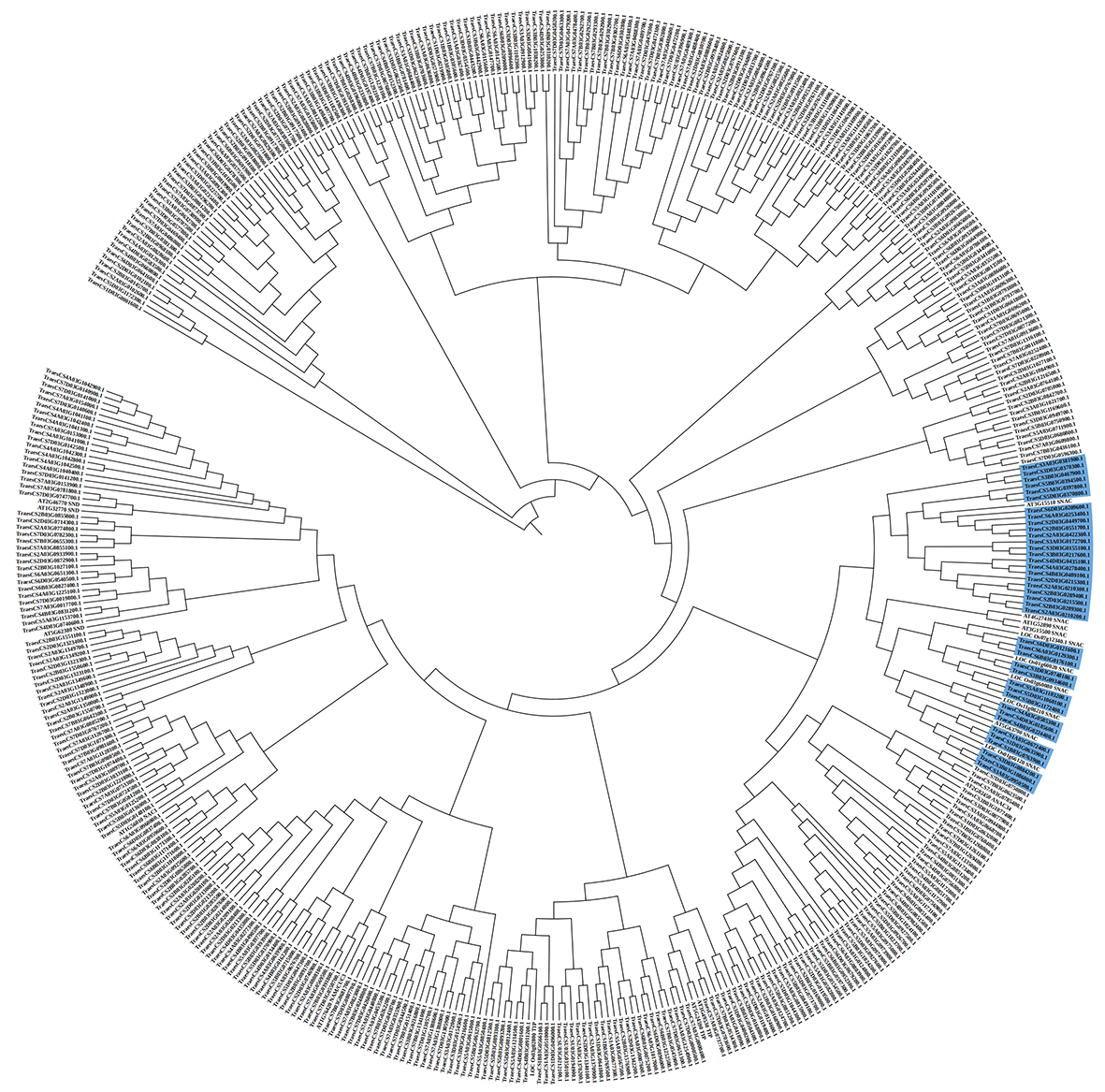

Supplement: Supplementary Figure 1 — The phylogenetic tree using 452 TaNACs and 12 NACs from Arabidopsis for TaSNAC identification. [file Image_1.TIF]

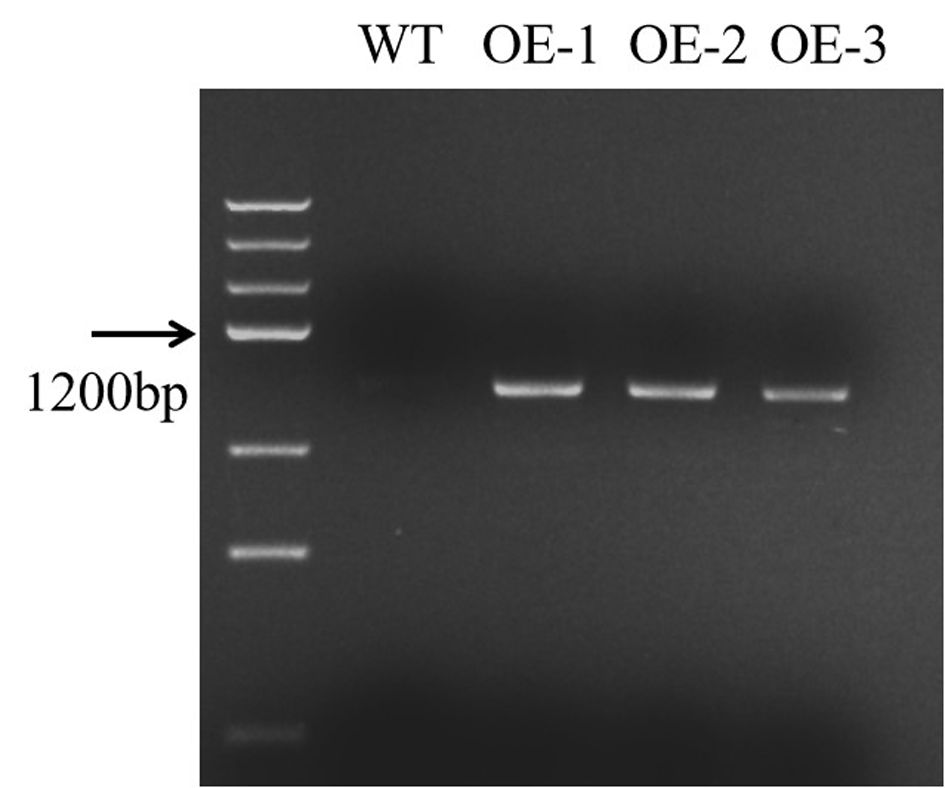

Supplement: Supplementary Figure 2 — Identification of TaSNAC4-3D overexpression plants using PCR amplification. [file Image_2.TIF]
